# Supplementary material for: The interactome of intact mitochondria by cross-linking mass spectrometry provides evidence for coexisting respiratory supercomplexes
Source: Mol Cell Proteomics. 2017 Dec 8;17(2):216–32. doi: 10.1074/mcp.RA117.000470 (PMC5795388; doi:10.1074/mcp.RA117.000470)
Supplement: Supplemental Data [file supp_RA117.000470_133922_0_supp_23978_fzffwf.zip › spectra_annotation/mito_DR_spectra_annotation/474-1-1-1-8-1.pdf]

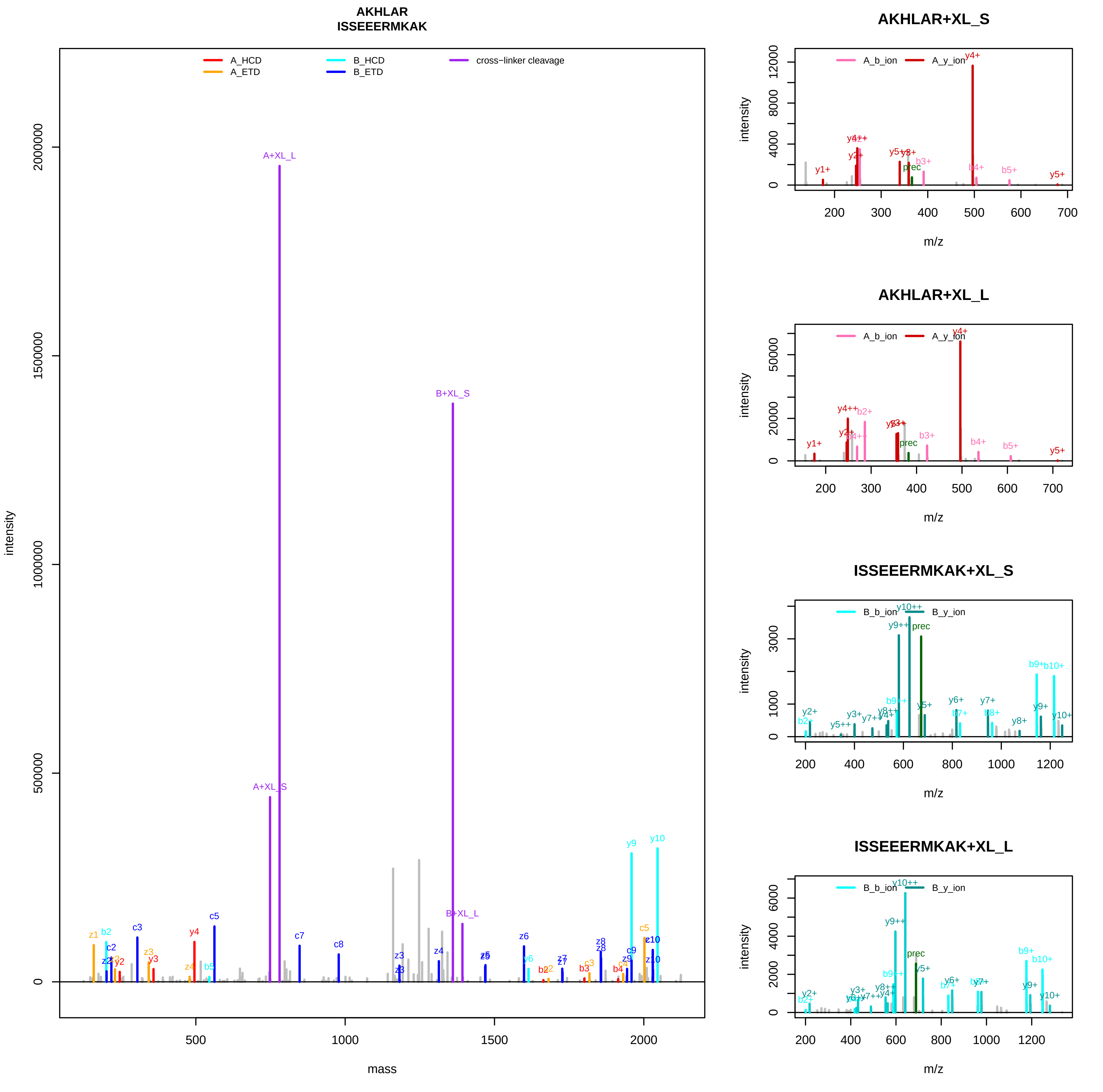

AKHLAR
ISSEEERMKAK
A_HCD
A_ETD
B_HCD
B_ETD
cross−linker cleavage
A+XL_L
B+XL_S
A+XL_S
B+XL_L
intensity
mass
0
500000
1000000
1500000
2000000
500
1000
1500
2000
z1 b2 c2 z2 c3 z3 y2 y3 y4 z4 b5 c5 c7 c8 z3 z4 z5 z6 y6 b2 z2 z7 c3 b3 z8 b4 c4 z9 c9 y9 c5 z10 y10
AKHLAR+XL_S
A_b_ion
A_y_ion
y4+ y4++ y2+ y1+ y5+ y3+ prec b3+ b4+ b5+
AKHLAR+XL_L
y4++ b2+ y2+ b4++ y1+ y5+ y3+ prec b3+ b4+ b5+
ISSEEERMKAK+XL_S
B_b_ion
B_y_ion
y10++ y9++ prec b9+ b10+ y2+ b2+ y3+ y5++ y7++ y4+ y8++ b9++ y5+ y6+ b7+ y7+ b8+ y8+ y9+ y10+
ISSEEERMKAK+XL_L
y10++ y9++ prec b9++ y5+ b9+ b10+ y2+ b2+ y3+ y6++ y7++ y4+ y8++ y6+ b7+ b8+ y7+ y9+ y10+
intensity
m/z
0
4000
8000
12000
20000
50000
1000
3000
2000
6000
200
300
400
500
600
700
800
1000
1200
